# Optimizing Gaussian Process Regression for Image Time Series Gap-Filling and Crop Monitoring

**DOI:** 10.3390/agronomy10050618

**Published:** 2020-04-27

**Authors:** Santiago Belda, Luca Pipia, Pablo Morcillo-Pallarés, Jochem Verrelst

**Affiliations:** Image Processing Laboratory (IPL), Parc Científic, University of Valencia, Paterna, 46980 Valencia, Spain

**Keywords:** Gaussian processes regression, time series, crop monitoring, Sentinel-2, phenology indicators, optimization

## Abstract

Image processing entered the era of artificial intelligence, and machine learning algorithms emerged as attractive alternatives for time series data processing. Satellite image time series processing enables crop phenology monitoring, such as the calculation of start and end of season. Among the promising algorithms, Gaussian process regression (GPR) proved to be a competitive time series gap-filling algorithm with the advantage of, as developed within a Bayesian framework, providing associated uncertainty estimates. Nevertheless, the processing of time series images becomes computationally inefficient in its standard per-pixel usage, mainly for GPR training rather than the fitting step. To mitigate this computational burden, we propose to substitute the per-pixel optimization step with the creation of a cropland-based precalculations for the GPR hyperparameters ***θ***. To demonstrate our approach hardly affects the accuracy in fitting, we used Sentinel-2 LAI time series over an agricultural region in Castile and Leon, North-West Spain. The performance of image reconstructions were compared against the standard per-pixel GPR time series processing. Results showed that accuracies were on the same order (RMSE 0.1767 vs. 0.1564 [m^2^/m^2^], 12% RMSE degradation) whereas processing time accelerated about 90 times. We further evaluated the alternative option of using the same hyperparameters for all the pixels within the complete scene. It led to similar overall accuracies over crop areas and computational performance. Crop phenology indicators were also calculated for the three different approaches and compared. Results showed analogous crop temporal patterns, with differences in start and end of growing season of no more than five days. To the benefit of crop monitoring applications, all the gap-filling and phenology indicators retrieval techniques have been implemented into the freely downloadable GUI toolbox DATimeS.

## 1 Introduction

Earth observation (EO) is used to monitor and assess continuously the status of, and changes in, natural and managed vegetated lands [1,2]. Today, a growing amount of EO data comes mainly from different airborne and satellite remote sensing observations. For instance, the Copernicus’ flagship for terrestrial EO, i.e., the Sentinel-2 (S2) constellation, provides free, full and open access optical data with very short revisit times (five days in mid-latitudes), high spatial resolution (10 m and 20 m), and good spectral resolution (10–180 nm) [3,4]. The usage of optical EO time series has opened the door to global-scale crop monitoring through their spectral properties using different kinds of vegetation indicators such as NDVI (Normalized Difference Vegetation Index) [5], LAI (Leaf Area Index, projected one-side leaf area per unit of ground area) [6] or fAPAR (Fraction of Absorbed Photosynthetically Active Radiation) [7]. To achieve that, the generation of continuous fields in time and space (i.e., gap-filling) starting from irregularly distributed data is of critical importance. However, these time series are associated with significant uncertainties and incomplete because of inadequate climatic conditions (e.g., clouds, snow and aerosols), and the long interval needed for the satellites to revisit and acquire data for the exact same location [8]. Consequently, robust gap-filling solutions are required for accurate crop phenology characterization [9–11]. A diversity of interpolation and fitting methods can perform this task (e.g., see review of [12]), but the difficulty lies in the choice of the one that successfully reconstruct vegetation indices and retrieve reliable phenology indicators such as dates of start and end of growing season (SOS and EOS, respectively), maximum peak, day of maximum value (DOM) (when the largest value per cycle occurs), amplitude (difference between the maximum and the average of the left and right minimum values per season), length of the season (LOS), etc. [13], which are narrowly related to essential sources of information including start of senescing, harvest day, productivity estimates, irrigation management, nutrient management, health management, yield prediction, and crop type mapping [14–16].

Artificial intelligence (AI) is a thriving field with many practical applications and active research topics (e.g., remote sensing). In the early days of AI, the field rapidly tackled and solved problems that are intellectually difficult for human beings but relatively straightforward for computers—Problems that can be described by a list of formal, mathematical rules [17]. Machine Learning (ML) can be defined as a subset of AI. In ML, machines have the ability to learn on their own without being explicitly programmed [18,19]. In the last decade, ML has attained outstanding results in estimating climate variables and related biogeophysical variables (e.g., LAI) from the acquired images at local and global scales [20–23].

Of specific interest to filling gaps in time series is the emergence of machine learning regression algorithms (MLRAs) which can serve as fitting functions. Among the multiple MLRA approaches currently available, the kernel-based methods developed in a Bayesian framework deserve special attention, such as Gaussian processes regression (GPR) [24]. Recent studies demonstrated the effectiveness of GPR for LAI time series gap-filling [25–27]. GPR carries out a non-parametric fitting developed in a Bayesian framework and provides uncertainty intervals along with the mean estimates [28]. This distinct feature, which is not shared by other machine learning algorithms, can open a unique source of information to assess the robustness of the predictions at various temporal scales. The entire procedure of learning a GPR model only relies on appropriate selection of the type of kernel and the hyperparameters involved in the estimation of input data covariance. Kernels contain our assumptions about the function we wish to learn and define the closeness and similarity between data points. Once a kernel is selected, the unknown hyperparameters of the kernel need to be learned from the training data [29]. This can be done by marginal likelihood maximization, attempting to minimize for example the squared prediction errors. Finally, inference of the hyperparameters and the weights for doing predictions can be performed by continuous evidence optimization. We will call this optimization procedure GPR training. Despite its clear strategic advantage, the most important shortcomings of this technique are their (1) high computational cost and (2) memory requirements [30], which grows cubically and quadratically with the number of training points, respectively [31,32]. This can become problematic in view of processing a large amount of data, such as in S2 time series tiles. Hence, strategies need to be developed on how to speed up the GPR processing while maintaining the superior performance in terms of accuracy.

In an attempt to optimize GPR time series processing, in this study we describe an efficient approach to reduce the overall computational burden involved in the hyperparameters optimization during training stage, and accelerate the gap-filling procedure at pixel level over multiple croplands.

The remainder of the paper is structured as follows. The GPR theory is described in Section 2. Section 3 outlines the data and the followed methodology. Section 4 provides the results. Discussion is presented in Section 5 whereas conclusions and future work lines are finally presented in Section 6.

## 2 Gaussian Process Regression

Standard Gaussian Process Regression (GPR) models are state-of-the-art statistical methods for regression and function approximation. In recent years, we have successfully applied GPRs for the retrieval of biophysical parameters from optical imagery, see [21–23,28,33–37]. GPR models yield not only predictions of the phenomenon to be characterized by means of a non-parametric modelling, but also an estimation of their uncertainty. A general introduction to GPR can be found in [22,24]. In the following we briefly review the standard GPR adapted to the general needs of this study.

Let $𝒟={\left\{t_{i},y_{i}\right\}}_{i=1}^{N}$ be a set of *N* pairs of a generic parameter *y_i_* extracted from data acquired at times *t_i_.* We use these pairs to learn a function *f* able to predict the parameter estimates at new times. Instead of assuming a parametric form for *f*, we start by assuming an additive noise model:

(1) $$y_{i}=f\left(t_{i}\right)+e_{i},\;e_{i}∼𝒩(0,\sigma_{n}^{2}),$$

where *t* ∈ ℝ, $\sigma_{n}^{2}$ is the noise variance and *f* (*t*) is the unknown (and nonparametric) latent function to be found. Defining **t** = [*t*_1_,…, *t_N_*]^⊺^, the GPR model assumes that *f* (**t**) is a Gaussian-distributed random vector with zero-mean and covariance matrix **K**(**t**, **t**), i.e., *f*(**t**) ~ *𝒩*(**0**, **K**). The elements *ij* of the covariance matrix are calculated by means of a kernel function *k*(*t_i_*, *t_j_*) encoding the similarity between input time points *t_i_* and *t_j_*. Various covariance functions (kernel functions), with associated kernel parameters ***θ*** (i.e., hyperparameters), can be employed in a GPR ([24,38]): Squared Exponential (SE), Matern 3/2, Matern 5/2 and Rational Quadratic (RQ), among others. The choice of the covariance function, and consequently of its hyperparameters, is called model selection.

In this study, we pay special attention to the most commonly employed SE covariance function (Equation (2)), which reflects our prior assumption that the function to be learned, i.e., the evolution of any vegetation descriptors in time, is smooth:

(2) $$k\left(t_{i},t_{j}\right)=\sigma_{f}^{2}\text{exp}\left(-\frac{1}{2l^{2}}{\left(t_{i}-t_{j}\right)}^{2}\right)+\sigma_{n}^{2}\delta_{ij}$$

where $\sigma_{f}^{2}$ > 0 is the signal variance, *l* > 0 is the length-scale, $\sigma_{n}^{2}$ >= 0 is the noise covariance and *δ_ij_* is a Kronecker delta which is 1 if i = j and zero otherwise. These free parameters $\theta =\left\{\sigma_{f}^{2},l,\sigma_{n}^{2}\right\}$ allow for flexible customization of the GPR for a wide variety of regression problems. They have the following interpretation:

- Length-scale *l* describes how smooth a function is. Small length-scale value means that function values can change quickly; large values characterize functions that change only slowly. *l* also determines how far we can reliably extrapolate from the training data.
- Signal variance $\sigma_{f}^{2}$ is a scaling factor. It determines variation of function values from their mean. Small value of $\sigma_{f}^{2}$ characterize functions that stay close to their mean value, larger values allow more variation. If $\sigma_{f}^{2}$ is too large, the modelled function will be free to chase outliers.
- Noise variance $\sigma_{n}^{2}$ is formally not a part of the covariance function itself. It is used by the Gaussian process model to allow for noise present in training data. This parameter specifies how much noise is expected to be present in the data.

The Bayesian framework allows us to estimate the distribution of f_*_ at the test point t_*_ conditioned on the training data, *p*(f_*_|*𝒟*, t_*_). According to the GPR formulation, *f*(t_*_) is normally distributed with mean and variance given by:

(3) $$\begin{array}{c} f\left(t_{*}\right)=\mu_{\text{GPR}}\left(t_{*}\right)=k_{*}^{⊤}{\left(K+\sigma_{n}^{2}I\right)}^{-1}y\\ \sigma_{f}^{2}\left(t_{*}\right)=\sigma_{\text{GPR}}^{2}\left(t_{*}\right)=c_{*}-k_{*}^{⊤}{\left(K+\sigma_{n}^{2}I\right)}^{-1}k_{*} \end{array}$$

where k_*_ = [*k*(t_*_, *t*_1_),…,*k*(t_*_, *t_N_*)]^⊺^ is an *N* × 1 vector, ***y*** = [*y*_1_,…,*y_N_*]^⊺^ and c_*_ = *k*(t_*_, t_*_) + $\sigma_{n}^{2}$.

For Gaussian process regression with Gaussian noise it is possible to obtain the probability of the data given the hyperparameters *p*(**y**|**t**, ***θ***) by marginalization over the function values *f* [24]. The log marginal likelihood is given by:

(4) $$\log p(y|t,\theta )=-\frac{1}{2}y^{T}{\left(K+\sigma_{n}^{2}I_{N}\right)}^{-1}y-\frac{1}{2}\log\left|K+\sigma_{n}^{2}I_{N}\right|-\frac{n}{2}\log2\pi$$

The first term in Equation (4) can be interpreted as a data-fit term, the second term is a complexity penalty and the last term is a normalizing constant. The derivatives of the log marginal likelihood with respect to the hyperparameters are given by:

(5) $$\frac{\partial }{\partial \theta_{j}}\log p(y|t,\theta )=-\frac{1}{2}tr\left(\left(\alpha \alpha^{T}-{\left(K+\sigma_{n}^{2}I_{N}\right)}_{y}^{-1}\right)\frac{\partial \left(K+\sigma_{n}^{2}I_{N}\right)}{\partial \theta_{j}}\right)$$

where *α* = **K**^−1^**y**. Any gradient-based optimization algorithm can be applied to Equation (5) to obtain the hyperparameters that maximize the marginal likelihood of a GPR. We will call this optimization procedure training the GPR [39,40].

## 3 Data and Methods

### 3.1 Data Description

**Figure 1:**
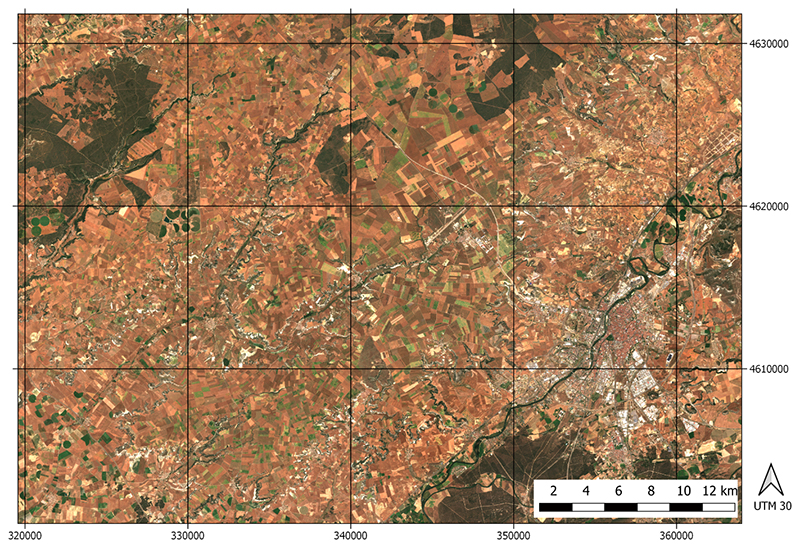
RGB image of the crop ROIs in Castile and Leon region, Northwest Iberian peninsula, from Sentinel 2 capture of 26 June 2016.

We prepared a demonstration case to assess the validity of the GPR time series processing. An agricultural region in Castile and Leon, in North-West of Spain, was chosen. The area shown in Figure 1 was selected as part of a wider validation region of SENSAGRI H2020 Project [21], for which a highly detailed land-cover map is yearly retrieved by using a random forest classifier on satellite imagery time series [41]. The classifier distinguishes between 50 specific crop types, being 35 of them arable crops, seven are irrigated crops and 8 are permanent crops [41]. The scene selected for the demonstration cases is mainly characterized by an intensive dryland agricultural system where the arable land comprises up to 80% of the available area.

For the experiments, we used green leaf area index (LAI) generated from atmospherically corrected S2 imagery using the GPR model developed in the framework of SENSAGRI project [21]. The S2 time series collection consists of 127 unevenly spaced and largely cloud-free acquisitions between November 2015 to September 2019.

### 3.2 Methodology

As opposed to other nonlinear machine learning methods, GPR has proven to be an attractive and effective tool for gap-filling of biophysical parameter time series [25–27] because the hyperparameters ***θ*** can be optimally set for each time series (one for each pixel in the area) with a single optimization procedure. A conventional approach adopted by most GPR users is a heuristic method, i.e., the optimization is repeated using several initial values generated randomly from a simple prior distribution. The final estimates of the hyperparameters are the ones with the largest likelihood values after convergence [24,42]. However, one of the main difficulties is the computational burden in estimating the final hyperparameters, and consequently the inverse of the covariance matrix in Equation (3), whose dimension grows proportionally with the number of training samples. To address such shortcoming and repetitive procedure for each pixel, we propose the use of precalculated hyperparameters to speed up the training stage of the GPR algorithm. The pursued experimental setup is detailed here:

1. **Crop type selection.** For each crop type found in the available dataset (i.e., wheat, corn, barley, sunflower, rape, pea, alfalfa, beet and potato), we randomly selected 100 parcels larger than 50 pixels.
2. **Hyperparameter optimization.** Hyperparameters were optimally determined by assessing individually each pixel, across the time series.
3. **Hyperparameter average.** In this step, we simply took the mean of the previously trained hyperparameters for each crop type. Additionally, we also computed a global average of the hyperparameters over all pixels within the randomly selected parcels (i.e., without any crop segregation).
4. **Time series prediction.** Subsequently, LAI-reconstructed time series were computed with different GPR model parameterizations, i.e., using the hyperparameters described in point 2 and 3.
5. **Statistical analysis for performance comparison.** In this step, we evaluated the performance of the different GPR models in terms of reconstruction (original vs. recontructed LAI time series) and processing time. The performance was assessed with the goodness-of-fit indicator root mean square error (RMSE), i.e., the lower the RMSE the better the reconstruction.
6. **Phenological metrics extraction.** Finally, we analyzed how the different GPR parametrizations (i.e., free vs. fixed hyperparameters) affect the estimation of phenological indicators derived from the reconstructed LAI time series. For determining when the seasons start and end, we used a percentage of the seasonal amplitude, defined between the base level and the maximum value for each individual season [27,43]. For easy visualization and interpretation, we calculated the SOS when the left part of the fitted curve reached a 20% of the seasonal amplitude, counted from the base level. The EOS was defined similarly, but using the right side of the curve.

With ambition to tackle these procedures in a streamlined way, the entire analysis was conducted in the so-called Decomposition and Analysis of Time Series Software (DATimeS) [27], a novel and generic in-house developed scientific time series toolbox. DATimeS is a stand-alone image processing GUI toolbox written in MATLAB. In short, DATimeS enables performing different advanced time series tasks for: (1) generating spatially continuous maps from discontinuous data using advanced MLRA (e.g., GPRs) and synergy of multiple sensors [26]; and (2) detecting heterogeneous spatial patterns of phenological indicators (i.e., crop key growth stages) throughout multiple seasons.

## 4 Results

### 4.1 Performance of GPR Models

The core part of this study is ascertaining how using precalculated hyperparameters optimized over crop areas affects the LAI estimates and performance of GPR models. Since the result from models obtained by performing the per-pixel hyperparameters optimization, here assumed as reference or “true” models, the accuracy of the different estimates can easily be compared. The hyperparameters averaged per crop are shown in Table 1.

Table 2 gives the RMSE between the LAI time series reconstructed by using the conventional per-pixel hyperparameters (***θ***_*pp*_) versus the ones calculated by using per-crop hyperparameters (***θ***_*pc*_) and global (all-crop) hyperparameters (***θ***_*gl*_). Regardless of which hyperparameters are used (either those computed using the same crop type or another specific crop type), the RMSE compared to the per-pixel hyperparameters is low, producing practically identical predictions. In the worst case scenario, the RMSE is hardly 0.196 [m^2^/m^2^]. Similar outcomes are achieved using ***θ***_*gl*_ (RMSE < 0.1 [m^2^/m^2^]). As expected, the most accurate results are mainly taking place when compared with the same crop type (bold numbers in Table 2).

An additional proof is given by the variation, in percentage, of the retrieved LAIs with respect to the benchmark (Table 3). This is calculated by dividing the RMSE estimated in Table 2 by the differences between the highest and lowest LAI values predicted with the reference GPR models. Once again, these results clearly illustrate that our approach based on using fixed averaged hyperparameters is almost insensitive to the crop-type selected for the time series gap-filling: defining a specific crop-type or averaging the hyperparameters of all crops led to very similar results, with final scores ranging from 1% to 9%.

It is well known that an incorrect choice of hyperparameters for GPR can significantly affect the performance of this method [44]. However, a more careful analysis of similarity between the time series of the same pixel obtained with the different approaches (Table 4) reveals significant high correlation values (>0.94). Accordingly, reliable LAI gap-filling can be carried out at the highest feasible accuracy independently of the crop type hyperparameter choice.

For easiest reference the variation in percentage of the S2-based LAI time series with respect to different GPR parametrizations are displayed in Table 5. It clearly shows how well different GPR models are progressing and where any weaknesses may lie. In general, the usage of per-pixel optimized hyperparameters resulted as the most accurate and robust models (bold numbers in Table 5) (e.g., 5.8% RMSE increase for barley). After analyzing different GPR parametrizations, it can be concluded that the accuracy does not change significantly with respect to the crop-type chosen for the optimization; they produce practically insignificant scatter for operation purposes (e.g., ranging from 5% to 13%). Also noteworthy is that some crops (e.g., wheat or barley) are more accurate than others (e.g., pea and sunflower), probably for the higher number of samples available for the former two classes.

Finally, we compared the computational time required to calculate GPR hyperparameters using the conventional per-pixel optimization approach compared with our proposed pre-calculated hyperparameter approach. The recorded processing time in Table 6 indicates that the proposed methodology outperforms the conventional strategy being about 90 times faster. Please note that for the estimation of the computational performance when precalculated hyperparameters are used, per-crop or global approaches are identical and a unique column accounts for both of them. Besides, the global model (i.e., GPR model computed by averaging the hyperparameters for each pixel time series, independently of the crop type) resulted as an optimum trade-off between quality and computational cost (with accuracy degradation between 6% and 11%, and maximum RMSE of 0.30 [m^2^/m^2^]). Special attention also deserves the last column in Tables 2, 3 and 5, where the variance for each row was estimated. This analysis shows that some crop types are more sensitive to the different parameterizations (e.g., potato, pea or sunflower is more sensitive to the parameterization than beet, wheat or barley).

### 4.2 Performance of Crop Phenology Characterization

Having outlined the potential of fixing hyperparameters, as a follow-up application we compared the phenological indicators retrieved from the LAI time series reconstructed by the proposed GPR parametrizations (see. Section 4.1). Since accurate spatiotemporally explicit knowledge of vegetation phenology is critical to understand the change trend of natural seasonal phenomena and serve for agricultural production and global change studies [45,46], this comparison becomes crucial to assess the sensitivity of the phenological parameters to the variations in the hyperparameters.

**Figure 2:**
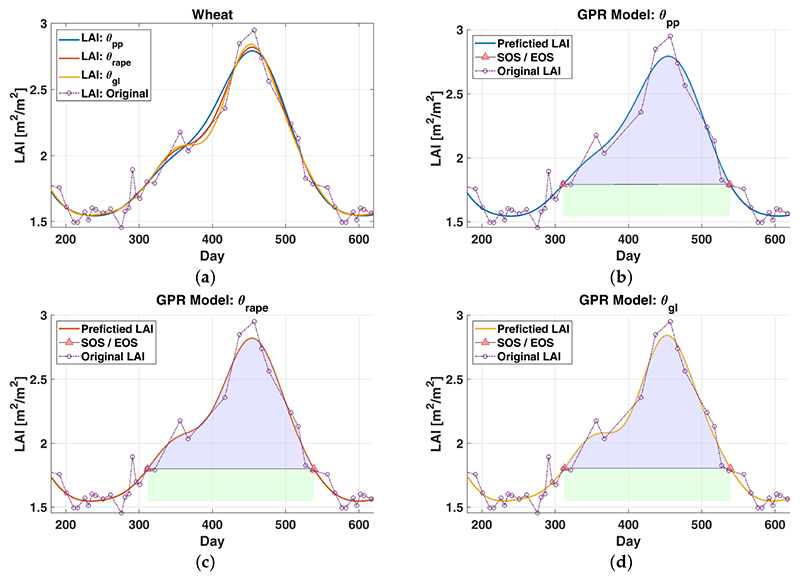
Modeling LAI time series of wheat by using different GPR parametrizations (***θ**_pp_,**θ**_Rape_,**θ**_gl_*) (Figure 2a) and automatic identification of some seasonal patterns (Figure 2b, 2c and 2d). The green and blue colors represent the area under the curve between SOS and EOS. For reasons of clarity the associated GPR uncertainties are not displayed. Counting of days starts from 1 January 2016.

**Figure 3:**
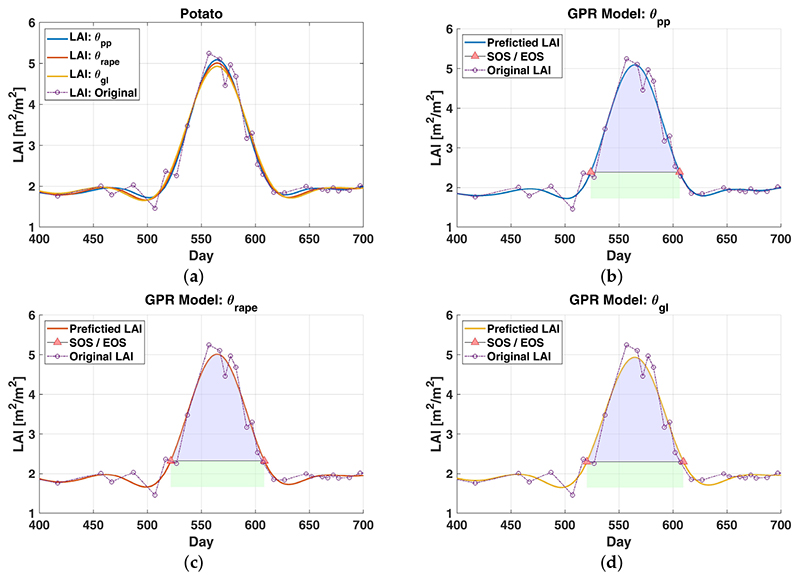
Modeling LAI time series of potato by using different GPR parametrizations ***θ**_pp_,**θ**_corn_,**θ**_gl_*) (Figure 3a) and automatic identification of some seasonal patterns (Figure 3b, 3c and 3d). The green and blue colors represent the area under the curve between SOS and EOS. For reasons of clarity the associated GPR uncertainties are not displayed. Counting of days starts from 1 January 2016.

As described in Section 3.2, DATimeS was used for the automatic identification of vegetation temporal patterns. To start with a simple example, let us focus primarily on two single pixels, wheat (Figure 2a) and potato (Figure 3a). Using the same scheme as above, their phenological events were estimated testing different LAI-reconstructed time series. In the former case, we used per-pixel optimized GPR models (***θ***_*pp*_). In the second test, hyperparameters were kept constant to a simple global average estimated by using all the pixels (***θ***_*gl*_). In the latter case, these parameters were defined similarly, but taking only the mean of different crop types, rape *(***θ***_*rape*_*) and corn (***θ***_*Corn*_).

The results obtained for the described experiment are shown graphically in Figures 2 and 3 and reported numerically in Table 7. For both cases (potato and wheat), we can see clearly that the temporal evolution of reconstructed LAI profiles offer similar performance among the use of conventional GPR models, where each crop presents comparable patterns throughout the different GPR parametrizations. For wheat, the dates of SOS/EOS/DOM determined with distinct GPR settings (***θ***_*pp*_,***θ***_*Rape*_,***θ***_*gl*_) differ slightly no more than 2 days. Apart from that, the seasonal amplitude and the maximum value for each individual LAI-reconstructed time series are insignificantly affected (LAI differences of about 0.1 [m^2^/m^2^]). Besides, the influence of different GPR settings on the seasonal integral (i.e., area under the curve between SOS and EOS) shows the biggest, but not significant, inconsistencies of about ≈8 [m^2^/m^2^d] (approximately 6%). For potato, similar conclusions can be drawn by comparing the interpolated LAI values using ***θ***_*pp*_,***θ***_*corn*_ and ***θ***_*gl*_.

Keeping the same scheme as before, a careful “global analysis: of phenological indicators was done by comparing the different pairs of reconstructed LAI time series (i.e., including all pixels). For numerical assessment, we calculated the mean absolute deviation (MAD) of the phenological metrics using the fixed ***θ*** approach regarding the ones derived from the conventional per-pixel optimization technique (Table 8). As before, fixing hyperparameters per crop type or per multiple crop area (i.e., global) cause no statistically significant amplitude and maximum value differences (LAI <0.1[m^2^/m^2^]). Concerning SOS/EOS, slight mean differences are observable of about 5 days. Consequently, it produces fluctuations in LOS of around 7 days. Once again, the seasonal integral presents the largest deviations (≈7 [m^2^/m^2^d]), corresponding to approximately 5%.

**Figure 4:**
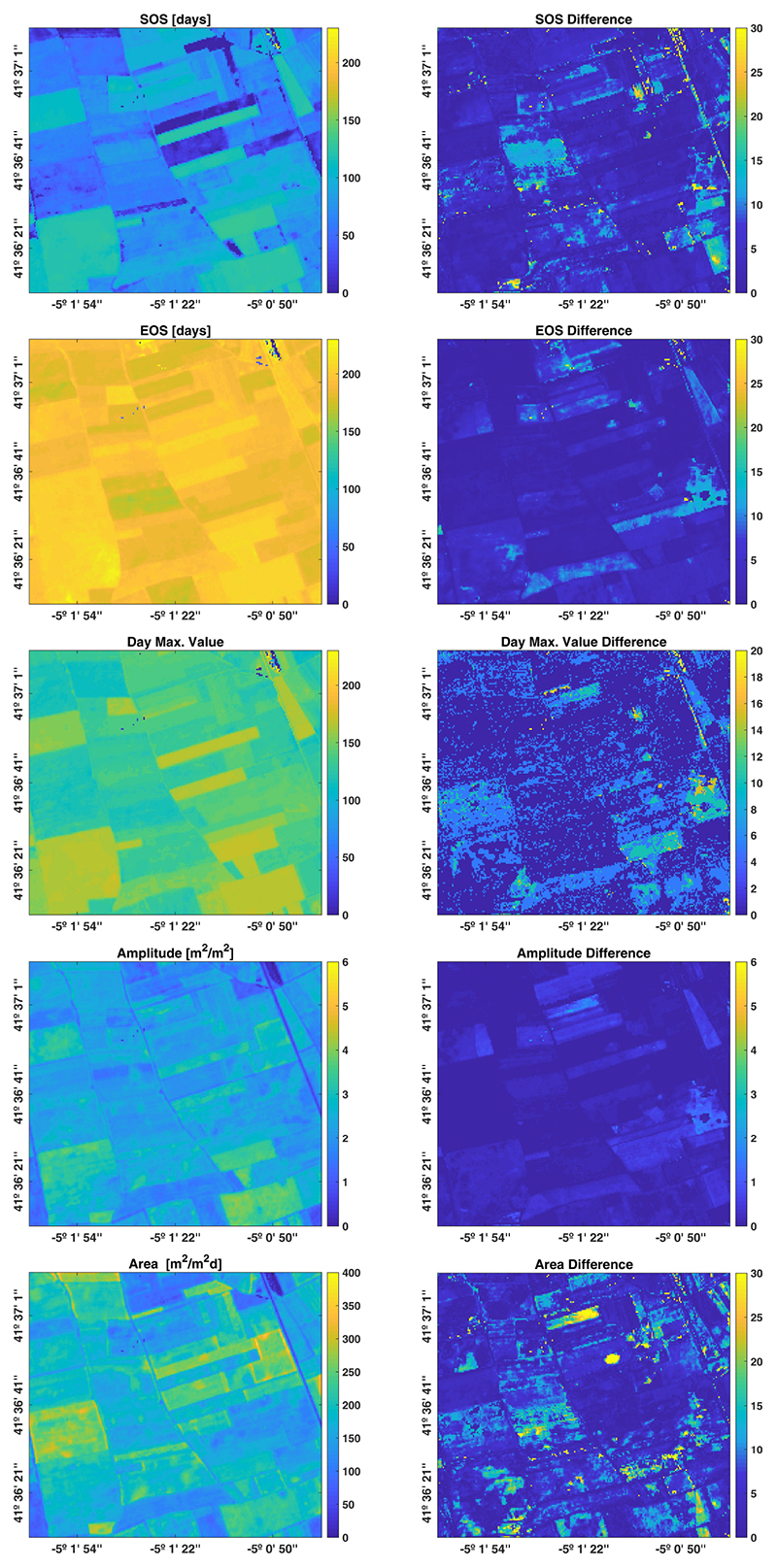
Phenological indicator maps estimated by using ***θ**_gl_* (**left** column) and their differences regarding ***θ**_pp_* (**right** column). Counting of days starts from 1 January 2017.

Finally, the spatial field-scale consistency of the result can be easily appreciated by visually inspecting the final maps in Figure 4 (left column), which were previously estimated by using the global set of hyperparameters ***θ***_*gl*_. In general, it shows good agreement in practically every phenological indicator. In the SOS map it can be clearly viewed that some crops started their growing season later. The EOS map is consistent as well, leading to homogeneous parcels in terms of length of season. Also, the day corresponding to the maximum value well resembles the pattern of the start of season. The differences w.r.t phenological maps derived from the conventional GPR approach clearly demonstrate a strong similarity, with RMSE differences of about 7 days and 0.22 [m^2^/m^2^] in SOS/EOS and amplitude, respectively (right panels in Figure 4). Based on these results, we can affirm that the reconstruction of multiple-seasons vegetation temporal patterns are quite insensitive to fixed hyperparameters optimized over either homogeneous or heterogeneous crop areas.

## 5 Discussion

GPR is a promising fitting method for gap-filling purposes. In two earlier comparison studies against alternative interpolation and curve fitting algorithms, GPR was evaluated as top-performing in accurately reconstructing time series images [26,27]. However, GPR is a kernel-based machine learning method and in its conventional usage goes along with a computational cost because for each pixel it goes through a training phase whereby the model hyperparameters ($l,\sigma_{f}^{2},\sigma_{n}^{2}$) are optimized. While processing time is negligible for a single pixel time series (i.e., in order to 0.1 s), when running pixel-by-pixel over images it accumulates to a long run-time. It makes this method impractical when aiming to process time series of complete Sentinel-2 tiles, which contains over 30 M pixels at 20 m resolution. Therefore, computationally efficient alternatives had to be sought that enables dealing with such big data.

As an efficient and fast solution, we proposed and evaluated whether the GPR ***θ*** hyperparameters can be precalculated per crop and kept fixed for the characterization of crop dynamics. With a Sentinel-2 demonstration case of LAI time series we showed that performance in terms of RMSE stays alike when comparing against the default per-pixel optimized setting. Hyperparameters can be kept fixed per crop type but also globally, i.e., for the heterogeneous crop area. Overall, their mean reconstruction accuracies in terms of RMSE are 0.1767 and 0.1792 [m^2^/m^2^], respectively, as opposed to 0.1564 [m^2^/m^2^] for the standard GPR estimations (i.e., 12% RMSE increase). At the same time the gain in processing time is up to 90 times faster. Altogether, these results suggest that optimizing the GPR hyperparameters ***θ*** over a limited subset of crop pixels, either homogeneous or heterogeneous, and then fixing their value for the whole crop area leads to a slight loss in accuracy, while gaining tremendously in run-time. We therefore believe that this method is a promising way forward in view of time series processing of large data, such as S2 tiles.

As an application to demonstrate the utility of the fixed ***θ*** approach, we used the two proposed strategies (per crop type and global) for time series reconstruction to enable subsequent calculation of phenology indicators. Results were alike as opposed to the standard GPR method with per-pixel hyperparameters. For instance, focusing on the global analysis summarized in Table 8, the maximum phenological pattern inconsistencies were never greater than 5 and 7 days for SOS/EOS and LOS, respectively. This again confirms that the hyperparameters can be safely kept fixed for the processing of agricultural areas.

Although GPR is a rather novel machine learning method that has hardly been applied to time series processing, some recent studies started exploring GPR in crop monitoring studies. An initial time series study was recently published by Campos-Taberner [35] where multitemporal LAI maps were processed from SPOT and Landsat data to monitor rice fields. It enabled identifying the occurrence of specific phenological phases such as green-up and maturation of the fields. In a follow-up study the GPR models were extended to Sentinel-2 and Sentinel-1 time series processing [47]. Further progress was achieved by Pipia [26], who used multi-output GPR models to fuse multiple data sources for improved spatiotemporal reconstruction of vegetation products such as LAI. This approach proved to be successful when fusing with radar data in case of persistent cloud cover and gaps become too long for accurate reconstruction based on optical data alone. Nevertheless, what all these GPR time series studies have in common is that they processed rather small agricultural regions, merely intended as demonstration cases. This is not surprising, given GPR’s per pixel training computational costs. In this respect, with the here proposed alternative, it may well become possible to overcome this limitation and process time series of complete tiles, meaning that more operational processing to the benefit of crop monitoring can become possible on cloud-computing platforms such as Google Earth Engine (GEE) or Amazon Web Service (AWS).

As a final remark, we are well aware that actors involved in crop monitoring activities may not be familiar with machine learning methods or how to run these methods. A key aspect in transferring new methods to a broader community implies easy access and easy use. To this end, we have implemented the here proposed method as an option into the DATimeS software toolbox [27]. DATimeS is a GUI toolbox that only requires a few essential steps such as (1) loading the satellite time series data, (2) selecting a region of interest if desired, (3) defining cloud mask and then (4) selecting the gap-filling fitting method, and finally (5) a gap-filling option. The user can choose either to fill solely the gaps due to cloud cover, but can also choose to reconstruct time series images according to a user-defined or fixed time step (e.g., every 10 days). When selecting GPR as fitting method, in version 1.1 the option has been added to fix hyperparameters, e.g., per crop type when a land cover map is provided. Fixing is done by asking the user for specific hyperparameters or by giving the possibility to use those derived from this study. In case no land cover map is available, standard GPR methodology can be applied. Finally, the option to compute a new set of precalculated optimum hyperparameters over specific mask-defined regions (to be fully processed or randomly sampled) or specific class from an available land cover map will be added to the Matlab-based GUI in the next DATimeS release.

Once having the gap-filling step completed, as a next step, the phenology indicators can be calculated, and all in an automated fashion. With these improvements in DATimeS, we are convinced that DATimeS can contribute to: (1) a wider familiarity of machine learning methods for time series processing, (2) easy tools of gap-filling and subsequent calculation of phenology indicators, and (3) ability to process big time series data, thanks to the here presented GPR alternatives. The toolbox can be freely downloaded at http://artmotoolbox.com.

## 6 Conclusions

Gaussian processes regression (GPR) emerged as a promising machine learning method for time series gap filling. However, the training on a per-pixel bases makes that this method is considerably more computationally expensive as opposed to standard interpolation fitting methods such as empirical smoothing methods and curve fitting methods. To mitigate its computational burden, in this work we evaluated whether the hyperparameters ***θ*** can be preoptimized over a reduced set of representative pixels and kept fixed over a more extended crop area using Sentinel-2 time series of LAI maps over a Spanish agricultural region. Our analysis led to the following main findings:

- For all tested crop fields, fixing the hyperparmeters led to LAI accuracies (RMSE) on the order of 0.1767 [m^2^/m^2^], as opposed to 0.1564 [m^2^/m^2^] for the standard GPR estimations. This suggests only a small loss in accuracy of around 12%.
- When further simplifying to fix to one hyperparameter for all crop types, the performance was only degraded between 2% and 7% compared to using the per-pixel optimization.
- Using both methodologies, the gain in processing speed is 90 times faster than the standard GPR estimations (i.e., 0.00111 vs. 0.1 sec, respectively).
- To demonstrate the validity of the optimization, phenology indicators were calculated based on the different GPR strategies. The final maps show the good quality of the proposed approach, with no statistically significant RMSE differences regarding the conventional GPR methodology (e.g., 7.27 days in EOS/SOS).

Altogether, the conducted experiments adequately demonstrated that with precalculating and fixing ***θ*** substantial gain in run-time can be achieved in time series reconstruction while maintaining the advantages of GPR, i.e., a high accuracy and provision of associated uncertainties. Given that cloud cover is a common yet undesired part of optical imagery, we are therefore convinced this simplification is a promising approach for time series gap-filling processing, which in turn is an essential requirement for precise calculation of crop phenology indicators. To the benefit of the community and to facilitate the usage of this simplified GPR approach for crop monitoring studies, the method has been implemented into the freely downloadable GUI software package DATimeS.

## Figures and Tables

**Table 1:** Averaged hyperparameters estimated using fixed crop-type and global apporaches.

	Wheat	Corn	Barley	Sunflower	Rape	Pea	Alfalfa	Beet	Potato	Global
log (1/*l*)	−3.9432	−3.6245	−3.6819	−3.6563	−3.8655	−3.2352	−3.6324	−3.7147	−3.4294	−3.6430
log (*σ_f_*)	−0.6151	−0.1381	−0.6275	−1.4275	−0.0032	−0.9412	−0.9359	0.2405	0.1128	−0.4817
log (*σ_n_*)	−2.0441	−1.5917	−2.0289	−2.1427	−1.3874	−2.1000	−1.8461	−1.0593	−1.4976	−1.7442

**Table 2:** Mean RMSE of the reconstructed LAI time series using the standard per-pixel hyperparameters optimization regarding the proposed methodology (i.e., precalculated per crop-type/global hyperparameters). Last column exhibits the variance in the RMSE. Units: [m^2^/m^2^].

Crop Type	Averaged Hyperparameters										Variance
	Wheat	Corn	Barley	Sunflower	Rape	Pea	Alfalfa	Beet	Potato	Global	
**Wheat**	**0.028**	0.032	0.030	0.034	0.027	0.048	0.030	0.028	0.044	0.030	0.007
**Corn**	0.085	**0.046**	0.050	0.080	0.072	0.050	0.063	0.055	0.046	0.049	0.015
**Barley**	0.052	0.036	**0.037**	0.051	0.046	0.043	0.043	0.039	0.040	0.037	0.006
**Sunflower**	0.068	0.054	0.056	**0.059**	0.064	0.047	0.056	0.057	0.050	0.055	0.006
**Rape**	0.086	0.086	0.083	0.090	**0.084**	0.104	0.084	0.082	0.101	0.083	0.008
**Pea**	0.120	0.084	0.090	0.106	0.110	**0.064**	0.096	0.095	0.070	0.089	0.017
**Alfalfa**	0.082	0.069	0.070	0.075	0.078	0.066	**0.071**	0.072	0.069	0.069	0.005
**Beet**	0.125	0.091	0.092	0.118	0.112	0.105	0.101	**0.095**	0.101	0.092	0.012
**Potato**	0.196	0.087	0.104	0.169	0.167	0.062	0.135	0.121	**0.059**	0.104	0.046

**Table 3:** Variation in percentage of LAI obtained with precalculated hyperparameters with respect to the benchmark, i.e., LAI values predicted with per-pixel optimization. The last column exhibits the variance in the percentage

Crop Type	Averaged Hyperparameters										Variance
	Wheat	Corn	Barley	Sunflower	Rape	Pea	Alfalfa	Beet	Potato	Global	
**Wheat**	1.796	2.113	1.950	2.237	1.749	3.106	1.979	1.842	2.869	1.951	0.463
**Corn**	3.231	1.731	1.883	3.050	2.746	1.891	2.380	2.075	1.749	1.874	0.560
**Barley**	3.085	2.141	2.201	3.039	2.740	2.557	2.529	2.307	2.391	2.197	0.342
**Sunflower**	**8.837**	6.962	7.196	7.650	**8.256**	6.070	7.242	7.395	6.502	7.079	0.800
**Rape**	3.126	3.106	3.017	3.264	3.032	3.762	3.037	2.975	3.673	3.002	0.286
**Pea**	**8.669**	6.110	6.489	7.678	7.968	4.642	6.982	6.851	5.072	6.428	1.243
**Alfalfa**	6.585	5.556	5.653	6.006	6.257	5.297	5.702	5.755	5.513	5.586	0.386
**Beet**	3.549	2.579	2.617	3.342	3.166	2.982	2.857	2.698	2.847	2.591	0.336
**Potato**	5.468	2.419	2.890	4.711	4.663	1.730	3.771	3.374	1.654	2.890	1.292

**Table 4:** Mean correlation analysis between the LAI time series estimated by precalculated and per-pixel optimized kernel hyperparameters (lowest values in bold).

Crop Type	Averaged Hyperparameters									
	Wheat	Corn	Barley	Sunflower	Rape	Pea	Alfalfa	Beet	Potato	Global
**Wheat**	0.996	0.996	0.996	0.997	0.997	0.993	0.997	0.997	0.994	0.996
**Corn**	0.993	0.998	0.997	0.995	0.995	0.997	0.997	0.997	0.998	0.997
**Barley**	0.991	0.994	0.994	0.993	0.992	0.992	0.994	0.994	0.993	0.994
**Sunflower**	**0.937**	0.959	0.956	0.950	**0.944**	0.966	0.955	0.954	0.964	0.957
**Rape**	0.993	0.994	0.994	0.994	0.993	0.991	0.994	0.994	0.992	0.995
**Pea**	**0.943**	0.968	0.965	0.956	0.951	0.978	0.961	0.962	0.976	0.965
**Alfalfa**	0.968	0.978	0.977	0.973	0.971	0.979	0.976	0.976	0.978	0.977
**Beet**	0.992	0.995	0.995	0.994	0.993	0.993	0.995	0.995	0.994	0.995
**Potato**	0.976	0.993	0.991	0.984	0.981	0.997	0.988	0.989	0.996	0.991

**Table 5:** Variation in percentage of LAI obtained with precalculated hyperparameters with respect to the original LAI time series (lowest values in bold). Last column exhibits the variance in the percentage.

Crop Type	Per-Pixel Hyperpar.	Averaged Hyperparameters										Variance
		Wheat	Corn	Barley	Sunflower	Rape	Pea	Alfalfa	Beet	Potato	Global	
**Wheat**	**6.269**	6.721	6.006	6.165	6.698	6.571	5.138	6.425	6.298	5.335	6.166	0.512
**Corn**	**5.817**	7.334	6.223	6.445	7.134	7.066	5.408	6.762	6.631	5.554	6.432	0.640
**Barley**	**5.763**	7.150	6.098	6.308	7.043	6.904	5.250	6.663	6.493	5.396	6.309	0.637
**Sunflower**	**9.284**	12.717	10.948	11.278	12.165	12.283	9.493	11.691	11.562	9.850	11.251	1.150
**Rape**	**6.905**	8.049	6.779	7.111	7.843	7.830	5.288	7.474	7.356	5.504	7.085	0.900
**Pea**	**5.809**	10.845	8.908	9.252	10.252	10.357	7.422	9.730	9.560	7.765	9.234	1.482
**Alfalfa**	**8.714**	11.136	9.626	9.935	10.792	10.810	8.098	10.352	10.202	8.517	9.920	1.002
**Beet**	**7.436**	8.881	7.601	7.857	8.618	8.585	6.537	8.217	8.074	6.749	7.844	0.745
**Potato**	**4.628**	7.952	5.863	6.197	7.380	7.389	4.895	6.752	6.521	5.035	6.190	1.091

**Table 6:** Processing time (minutes) using the standard per-pixel hyperparameters optimization vs. the proposed methodology (i.e., precalculated per crop-type/global hyperparameters). Computer specifications: CPU i7-8700k @ 3.7 Ghz with 32 gb of RAM, running under windows 10—Matlab 2018b.

Crop Type	No. of Pixels	Time (m)		
		*θ_pp_*	[*θ_pc_,θ_gl_*]	Ratio
**Wheat**	62,482	104.136	1.145	90.95
**Corn**	36,065	60.108	0.661	90.93
**Barley**	44,154	73.590	0.809	9.,96
**Sunflower**	29,463	49.105	0.540	90.94
**Rape**	23,467	39.111	0.430	90.96
**Pea**	14,726	24.543	0.269	91.24
**Alfalfa**	21,683	36.138	0.397	91.03
**Beet**	16,466	27.443	0.301	91.17
**Potato**	14,337	23.895	0.262	91.20
**Total**	262,843	438.069	4.814	-

**Table 7:** Automatic identification of some seasonal patterns computed in DATimeS by using the reconstructed LAI curves shown in Figures 2 and 3. Units: days for SOS, EOS, LOS, DOM; [m^2^/m^2^] for max value and amplitude; [m^2^/m^2^d] for blue and green area

	Wheat			Potato		
	*θ_pp_*	*θ_Rape_*	*θ_g_*	*θ_pp_*	*θ_Corn_*	*θ_g_*
**SOS**	311	311	313	524	522	520
**EOS**	538	538	539	606	608	610
**LOS**	227	227	226	82	87	89
**DOM**	454	455	453	565	565	565
**Max Value**	2.79	2.80	2.84	5.09	5.01	4.93
**Blue Area**	115.01	113.08	107.30	129.70	135.62	137.16
**Green Area**	56.74	57.07	58.44	55.51	58.02	58.66
**Amplitude**	1.25	1.26	1.29	3.37	3.35	3.28

**Table 8:** Mean absolute deviation (MAD) of phenological metrics derived from the predicted LAI using real GPR models regarding different GPR parametrizations (i.e., using hyperparameter mean disaggregated by crop types and global hyperparameter average). Units: days for SOS, EOS, LOS, DOM; [m^2^/m^2^] for max value and amplitude; [m^2^/m^2^d] for blue and green area.

*θ*	SOS	EOS	LOS	DOM	Max Value	Blue Area	Green Area	Amp
**Crop Mean**	2.76 ± 4.9	3.47 ± 3.6	5.37 ± 10.6	3.49 ± 8.9	0.07 ± 0.1	5.37 ± 10.2	3.19 ± 5.3	0.09 ± 0.1
**Global Mean**	4.60 ± 8.5	4.99 ± 6.0	7.58 ± 11.2	4.66 ± 8.4	0.09 ± 0.1	6.69 ± 10.1	4.02 ± 6.2	0.12 ± 0.1
